# Pollution Characteristics and Possible Sources of Seldom Monitored Trace Elements in Surface Sediments Collected from Three Gorges Reservoir, China

**DOI:** 10.1155/2014/170639

**Published:** 2014-07-17

**Authors:** Bo Gao, Xin Wei, Huaidong Zhou, Jin Lu, Hong Hao, Xiaohong Wan

**Affiliations:** ^1^State Key Laboratory of Simulation and Regulation of Water Cycle in River Basin, China Institute of Water Resources and Hydropower Research, Beijing 100038, China; ^2^Department of Water Environment, China Institute of Water Resources and Hydropower Research, Beijing 100038, China; ^3^State Key Laboratory of Water Environment Simulation, School of Environment, Beijing Normal University, Beijing 100875, China

## Abstract

A geochemical study of Three Gorges Reservoir (TGR) sediments was carried out to analyze the concentrations, distribution, accumulation, and potential sources of the seldom monitored trace elements (SMTEs). The mean concentrations of Li, B, Be, Bi, V, Co, Ga, Sn, Sb, and Tl were 47.08, 2.47, 59.15, 0.50, 119.20, 17.83, 30.31, 3.25, 4.14, and 0.58 mg/kg, respectively. The concentrations of total SMTEs, together with their spatial distribution, showed that the SMTEs were mainly due to anthropogenic inputs in the region of TGR. The assessment by Geoaccumulation Index indicates that Tl, Be, V, Co, and Fe are at the unpolluted level; Bi, Li, Ga, and Sn were at the “uncontaminated to moderately contaminated” level. However, B was classified as “moderately contaminated” level and Sb was ranked as “strongly contaminated” level. The pollution level of the SMTEs is Sb > B > Bi > Li > Ga > Sn > Tl > Be > V > Co > Fe. The results of Correlation Analysis and Principal Component Analysis indicated Be, V, Co, Ga, Sn, Tl, Bi, and Fe in sediments have a natural source. B and Li were positively correlated with each other and mainly attributed into similar anthropogenic input. In addition, Sb has less relationship with other SMTEs, indicating that Sb has another kind of anthropogenic source.

## 1. Introduction

River sediment is both the source and sink of the heavy metals in water environment. It is also one of the most important media in water environment to assess the contamination level of aquatic ecosystems [1]. Up to now, the concentrations, accumulation, spatial distributions, sources, and ecological assessment of heavy metal pollution resulting from commonly monitored trace elements (i.e., As, Cd, Cr, Cu, Pb, and Hg) in river sediments have been deeply investigated [2, 3]. However, increasing industrial use of seldom monitored trace elements (SMTEs: Li, B, Be, Bi, V, Co, Ga, Sn, Sb, and Tl) would in theory lead to increased environmental concentrations. In addition, because of their bioavailability, toxicity, persistence, and nondegradability in the environment [4–10], SMTEs should be of global interest and be much accounted for; but there is little information on geochemical behaviors of SMTEs in river sediments. The knowledge of the concentrations, distributions, and enrichment of SMTEs in sediments plays a key role in tracing SMTEs sources and assessing the potential ecological risks of SMTEs in aquatic systems [7, 11].

The TGR area is located in China at west of Hubei Province and middle east of Chongqing city (28°32′–31°44′N and 105°44′–111°39′E), covering an overall area of 58, 000 km^2^ and including totally 20 districts and counties (cities). The reservoir waters and their fringe areas are generally called the TGR area of the Yangtze River. In fact, TGR is the largest hydroelectric project ever built in China, as well as in the world. The TGR plays acritically important role in economic development and national drinking water safety. In recent years, the researchers investigated the concentrations and distribution of the commonly monitored trace elements in sediments [2, 12], agricultural soils [13, 14], and aquatic organisms [15] in the region of TGR. However, little concern was arisen to the SMTEs in the aquatic environment. Until now, the concentrations and spatial distribution of SMTEs in sediments of TGR have not been detailedly reported. In fact, as an important category of pollutes in the water environment, a large quantity of industrial and mining activities in TGR may lead to a considerable the SMTEs pollution in this region. The primary objectives of this study were (1) to provide basic information of the concentration levels and spatial distribution of the SMTEs in sediments of TGR after the submergence, (2) to perform pollution assessment of SMTEs in sediments using the Geoaccumulation Index (*I*_geo_), and (3) to investigate the possible sources of SMTEs in sediments of TGR. It is hoped that this study will not only provide valuable information about SMTEs pollution in TGR after the submergence, but also present a scientific perspective of the environmental effects of the SMTEs in large reservoirs so that further regulatory and environmental attention can be drawn to the issue.

## 2. Material and Methods

### 2.1. Sampling of Sediments

**Figure 1:**
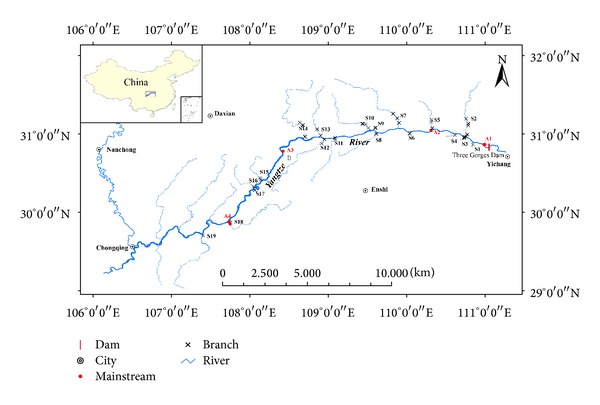
Location of sampling sites in TGR, China.

Seventy-three surface sediment samples were collected from the mainstream and major tributaries in TGR in March 2010 after the submergence period. The sampling sites were described within the zone (Figure 1). Four sampling sites were selected from mainstream (A1~A4) and sixty-nine sediment samples were collected from nineteen tributaries (S1 to S19) in TGR. About 2 to 5 sediment samples were collected for each tributary (mainly distributed in up, middle, and down area of tributaries). At each sampling site, sediment samples were taken using a plastic trowel near the middle of the flow of the stream. About 1 kg of sediment was collected into clean polyethylene bags and treated immediately after returning to the laboratory. The sediment samples were wet sieved through an acid-cleaned 63 *μ*m mesh nylon sieve in order to obtain the chemically active material, dried at 40°C (24 h) to constant weight, and ground in an agate mortar in order to ensure sample homogeneity.

### 2.2. Analytical Methods

All chemical treatments were in the ultraclean laboratory, and all reagents were of high purity grade. Total SMTEs concentrations in sediments were measured using established method [8]. Briefly, a mass of 40 mg of dry sample was weighed and dissolved into 10 mL Teflon bombs. About 4 mL concentrated HNO_3_ + 0.4 mL concentrated H_2_O_2 _were added to the samples and were left on a hot plate for one day. This step was to remove organic materials from sediment samples. The samples were then taken to dryness at 120°C. The residue was dissolved in 1.5 mL HNO_3_ + 1.5 mL HF of sample. After 20 min ultrasonic treatment, these samples were taken into sealed bomb and were placed in an oven at 190°C for 48 h. This procedure resulted in clear solutions for sediments. After evaporation at 120°C, samples were dissolved in 1% HNO_3_ (v : v). SMTEs concentrations of Li, B, Be, Bi, V, Co, Ga, Sn, Sb, and Tl were measured by inductively coupled plasma-mass spectrometry (ICP-MS, Perkin Elmer Elan DRC-e). The quality controls for the strong acid digestion method included reagent blanks, duplicate samples, and standard reference materials. The QA/QC results show no sign of contamination in all the analyses. The accuracy of the analytical procedures employed for the analysis of the SMTEs in surface sediments was checked using the certified reference material of China stream sediment (GSD-12, GBW07312), obtaining good agreements with the certified values (Table 1).

### 2.3. Index of Geoaccumulation (*I*_geo_)

The Geoaccumulation Index (*I*_geo_) introduced by Müller [16] was also used to assess SMTEs pollution in sediments of TGR. Geoaccumulation Index is expressed as follows:

(1) $$\begin{array}{c} I_{\text{geo}}={\text{Log}}_{2}\left(\frac{C_{n}}{1.5B_{n}}\right), \end{array}$$

where *C*_*n*_ is the measured concentration of trace metal (*n*) in the sediment, *B*_*n*_ is the geochemical background value [17] of element *n*, and 1.5 is the background matrix correction factor due to lithogenic effects. The Geoaccumulation Index includes seven grades from Class 0 (*I*_geo_ ≤ 0) to Class 6 (*I*_geo_ > 5). The Class 6 reflects at least 100-fold enrichment above the background values. Based on the *I*_geo_ data and Muller's Geoaccumulation Indexes, the contamination level with respect to each metal of sediment samples is ranked in Table 2.

## 3. Results and Discussion

### 3.1. Concentrations of SMTEs

SMTEs concentrations and statistics results in sediments of TGR are summarized in Table 2. SMTEs concentrations in background values and stream sediments in other important rivers of China are also presented in Table 2. As can be seen, the average concentrations of these SMTEs were ranked: V > B > Li > Ga > Co > Sb > Sn > Be > Tl > Bi. In general, the mean concentrations of SMTEs in TGR were slightly higher than those background values in stream sediments [18] and soils in China [19], indicating that the TGR sediments may be polluted by these SMTEs from anthropogenic sources. Comparing with those background values in this region, the average concentrations of Li, Be, Bi, V, Co, Ga, Sn, Sb, and Tl in TGR sediments were relatively higher than those of background values in Yangtze River, except for B and Sn [20]. Among these SMTEs, the Sb concentration is 5-fold higher than those of background value in Yangtze River, indicating the occurrence of an anthropogenic source of Sb. In fact, the SMTEs concentrations were also those concentrations in other rivers (Yellow River and Pearl River) in China. In addition, higher values of relative standard deviation (RSD > 40%) of Sb concentrations in sediments also suggested that the Sb sources were relatively complicated and anthropogenic inputs were possible to be the main source of the SMTEs (Table 3).

### 3.2. Distribution of SMTEs

**Figure 2:**
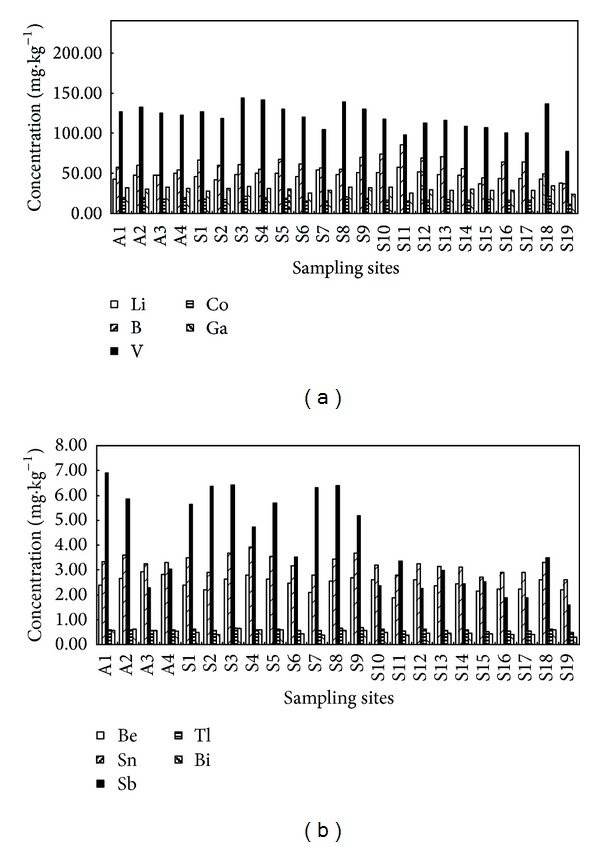
Spatial distribution of SMTEs in sediments from TGR tributaries.

Spatial distribution of the SMTEs in surface sediments of TGR is shown in Figure 2. In general, the SMTEs concentrations have an increasing tendency from upstream to downstream for the mainstream of TGR (from A4 to A1), especially for Sb. Among all the sediments, the highest Sb concentrations were found in the downstream (A1) in TGR. A large amount of anthropogenic activities in TGR area may explain this surprising enrichment of Sb, especially for mining activities and geological condition in upstream of TGR. In fact, the wastewater having the high concentrations Sb entered into the TGR and the SMTEs in water were absorbed into the suspended particles and eventually deposited into downstream with the water flow.

**Figure 3:**
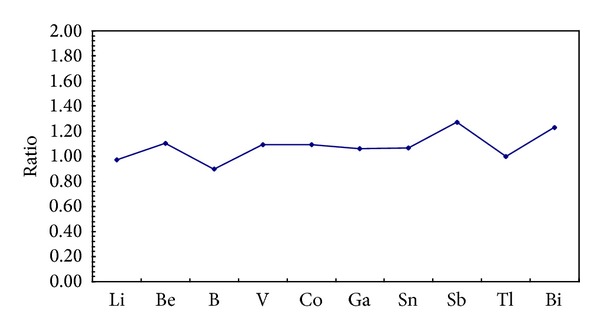
SMTEs concentration ratios in sediments between mainstream and tributaries.

The mean concentrations ratios of SMTEs between mainstream and tributaries were calculated and showed in Figure 3. In general, SMTEs concentrations in sediments collected from TGR mainstream were slightly higher than those values in tributaries, except for Li, B, and Tl (Figure 3). For tributaries, the concentration of SMTEs had an increasing tendency from upstream to downstream (S19 to S1), except for sampling site S18. In the sampling site S18, the SMTEs concentrations were relatively high among the sampling sites in upstream of TGR, especially for V, Co, Ga, Bi, and Fe. Because these SMTEs are known as the basic elements coming from natural geological sources, the high concentrations of V, Co, Ga, Bi, and Fe in S18 may be attributed into the relatively high environmental background in this region. Our results also showed that higher concentrations of Sb and Sn were also frequently detected in the downstream of TGR (Figure 3).

### 3.3. Pollution Assessment

Based on the *I*_geo_ data and Geoaccumulation Indexes, the results of the calculated *I*_geo_ values with respect to SMTEs are presented in Table 4. In general, the results of the average *I*_geo_ values are 0.65 for Li, −0.78 for Be, 1.99 for B, −0.78 for V, −1.09 for Co, 0.41 for Ga, 0.09 for Sn, 3.60 for Sb, −0.21 for Tl, 0.92 for Bi, and −1.29 for Fe, respectively. The order of average *I*_geo_ values in TGR sediments was Sb > B > Bi > Li > Ga> Sn > Tl > Be > V > Co > Fe. In general, the average *I*_geo_ values of Tl, Be, V, Co, and Fe are less than zero (*I*_geo_ ≤ 0), indicating that TGR sediments are unpolluted with respect to these five SMTEs. However, the average *I*_geo_ of Sb had the highest value and was ranked as “strongly contaminated” level (Class 4), indicating that TGR had significant accumulation of Sb apparently from anthropogenic sources. In fact, Sb was ranked as “extremely contaminated” in several sampling sites, including sampling sites S1, S2, S3, S5, S7, S8, and S9. In addition, B was classified as Class 2 which indicated the sediment quality as “moderately contaminated.” Bi, Li, Ga, and Sn were ranked in Class 1, standing for “uncontaminated to moderately contaminated” level in TGR sediments.

### 3.4. Origins of the SMTEs in Sediments

TGR receives a variety of inputs of heavy metals from nature and anthropogenic natural sources according to the high metal concentrations in sediments. In a further attempt to assess the sources responsible for the observed pollution levels, Correlation Analysis and Principal Component Analysis (PCA) were applied to data set of total concentrations of surface sediments in TGR. Correlation Analysis results (Table 5) indicated that Be, V, Co, Ga, Sn, and Fe have significant correlations with each other, suggesting that they had the same source. However, nonsignificant correlations of Sb with other ten elements (Li, Be, B, V, Co, Ga, Sn, Tl, Bi, and Fe) may be possibly due to different sources and have external inputs in sediments. In addition, the correlation results also showed that Li, B, and Tl were also positively correlated with each other, demonstrating a common source in sediments.

The method of the PCA is a multivariate statistical technique that can be employed to reduce the dimensionality of a data set while attempting to preserve the relationships present in the original data and was used to assess trace metal behavior in the aquatic system [8, 22, 23]. The rotated component matrixes of the PCA of SMTEs are presented in Table 6. The results of the PCA indicated that Li, Be, B, V, Co, Ga, Sn, Sb, Tl, Bi, and Fe concentrations could be reduced to three components (PC1, PC2, and PC3), which accounted for 76.83% of the total variance for all the data.

Firstly, the high loading of Be, V, Co, Ga, Sn, Tl, Bi, and Fe explained about 49.23% of the total variance. The concentrations of these elements were close to the background. Furthermore, Fe, Be, and V are generally named as the elements from the natural geological environment and Co, Ga, Sn, Tl, and Bi have a good correlation with Fe, Be, and V. Consequently, this factor can be identified as “natural factor.” The second component (PC2), which explains 16.71% of the total variance, appears to represent an “anthropogenic factor,” as it is strongly loaded with the elements of Li and B. In fact, TGR area is located in the Chongqing and Hubei Province and there is a high density of population. The anthropogenic activities may have an obvious impact on these two elements (B and Li) in sediments in TGR area. B is a common element in detergent and the detergent is frequently used in daily life such as washing powder. Therefore, the domestic wastewater including detergents may be one of the main sources of B and Li in the sediments. In fact, the high concentration of B is a distinct marker of household waste water. On one hand, the household wastewater including B is discharged directly into the river in rural areas. On the other hand, B in the water is also difficult to remove through simple water treatment procedures. Thirdly, the results of the PCA displayed that Sb has less association with other SMTEs and it is an independent component (PC3), indicating that Sb has another kind of anthropogenic source. Usually, Sb is a byproduct in mining activities. In this region, mining activities and metal refining activities in the upper region of TGR area may be responsible for these excess Sb.

## 4. Conclusions

Our investigation of SMTEs, such as Li, Be, B, V, Co, Ga, Sn, Sb, Tl, and Bi in sediment samples in TGR and its tributaries, observes that the mean concentrations of Sb in sediments of TGR were obviously higher than those in stream sediments of China and previous results, indicating the occurrence of an anthropogenic source of the SMTEs. The assessment by Geoaccumulation Index indicated that Tl, Be, V, Co, and Fe were at the unpolluted level; Bi, Li, Ga, and Sn were at the “uncontaminated to moderately contaminated” level, B was at the “moderately contaminated” level, and Sb was at the “strongly contaminated” level. The pollution level of the SMTEs is Sb > B > Bi > Li > Ga > Sn > Tl > Be > V > Co > Fe. The results of Correlation Analysis and PCA indicated that B and Li were positively correlated with each other, demonstrating a common source in sediments. In addition, our results indicated that the sediments in TGR have been severely contaminated by Sb and it has different anthropogenic sources among SMTEs. These results will provide fundamental pollution information of the SMTEs in TGR. Further research is required to investigate the behaviors and fates of SMTEs in river sediments and evaluate the bioavailability, toxicity, and ecological risks in water environment.

## Figures and Tables

**Table 1:** GSD-12 certified values, analytical values, and recovery.

Element	Analytical value (mg*·*kg^−1^)	Certified values (mg*·*kg^−1^)	Recovery (%)
Li	12.98	13.0	99.8
Be	0.63	0.9	86.7
Bi	0.35	0.38	92.1
B	27.28	26	104.9
V	103.95	107	97.1
Co	14.97	15.3	97.8
Ga	7.00	6.4	109.4
Sn	1.24	1.4	88.6
Sb	6.39	6.3	101.4
Tl	0.19	0.21	90.5

**Table 2:** Description of sediment quality.

*I*_geo_ rank	*I*_geo_	Pollution level
6	>5	Extremely contaminated
5	4~5	Strongly to extremely strongly contaminated
4	3~4	Strongly contaminated
3	2~3	Moderately to strongly contaminated
2	1~2	Moderately contaminated
1	0~1	Uncontaminated to moderately contaminated
0	<0	Uncontaminated

**Table 3:** Statistics results of SMTEs concentrations in sediments of TGR (mg*·*kg^−1^).

Statistic	Li	Be	B	Bi	V	Co	Ga	Sn	Sb	Tl	References
Min	37.30	1.89	37.31	0.30	77.47	11.30	24.24	2.60	1.59	0.48	This study
Max	54.03	2.83	73.74	0.69	143.55	21.53	34.53	3.90	6.90	0.67	This study
Mean	47.08	2.47	59.15	0.50	119.20	17.83	30.31	3.25	4.14	0.58	This study
S.D.	4.88	0.26	11.41	0.10	15.86	2.27	2.81	0.36	1.76	0.04	This study
RSD (%)	10.37	10.39	19.28	19.84	13.31	12.71	9.26	10.95	42.48	7.26	This study
Sediments in China	32	2.2	48	0.39	80	12	—	3.4	0.96	—	[18]
Soils in China	32	2.0	48	0.37	82	13	17.5	2.6	1.2	0.62	[19]
Crust	20	2.8	10	0.17	135	25	15	2	0.2	0.45	[21]
Yangtze River	43	1.9	63	0.42	97	17	16	3.5	0.83	0.49	[18]
Yellow River	23	1.7	52	0.13	60	9	11	2.5	0.62	0.45	[18]
Pearl River	40.1	—	—	5.02	113	16.2	13.1	1.46	—	—	[20]
Beijing River	—	—	—	—	28.56	9.64	17.25	85.61	38.98	—	[8]

**Table 4:** *I*_geo_values of SMTEs in sediments of Three Gorges Reservoir.

Station	Li	Be	B	V	Co	Ga	Sn	Sb	Tl	Bi	Fe
S1	0.62	−0.81	2.16	−0.68	−1.05	0.32	0.22	4.23	−0.16	0.93	−1.33
S2	0.49	−0.93	2.00	−0.78	−1.15	0.47	−0.04	4.41	−0.23	0.69	−1.24
S3	0.70	−0.67	2.02	−0.50	−0.80	0.58	0.29	4.42	−0.08	1.34	−0.92
S4	0.73	−0.58	1.87	−0.52	−0.92	0.47	0.38	3.98	−0.21	1.23	−1.07
S5	0.75	−0.68	2.18	−0.64	−0.96	0.45	0.24	4.24	−0.14	1.18	−1.32
S6	0.61	−0.76	2.04	−0.75	−1.24	0.17	0.08	3.55	−0.26	0.73	−1.34
S7	0.85	−1.01	1.91	−0.96	−1.26	0.35	−0.10	4.40	−0.23	0.59	−1.34
S8	0.69	−0.73	1.87	−0.55	−0.89	0.55	0.19	4.41	−0.07	1.11	−1.02
S9	0.77	−0.64	2.21	−0.64	−0.97	0.52	0.29	4.11	0.00	1.18	−1.65
S10	0.75	−0.69	2.30	−0.79	−1.19	0.54	0.09	2.97	−0.12	0.93	−1.38
S11	0.94	−1.15	2.51	−1.06	−1.26	0.18	−0.10	3.48	−0.30	0.60	−1.44
S12	0.78	−0.69	2.20	−0.85	−1.21	0.38	0.11	2.90	−0.13	0.84	−1.28
S13	0.68	−0.84	2.24	−0.80	−1.12	0.35	0.06	3.31	−0.23	0.83	−1.35
S14	0.67	−0.78	1.91	−0.90	−1.18	0.44	0.05	3.03	−0.20	0.81	−1.30
S15	0.31	−0.97	1.55	−0.92	−1.10	0.34	−0.14	3.07	−0.38	0.71	−1.25
S16	0.54	−0.91	2.10	−1.01	−1.19	0.36	−0.05	2.65	−0.31	0.68	−1.38
S17	0.54	−0.91	2.10	−1.01	−1.19	0.36	−0.05	2.65	−0.31	0.68	−1.38
S18	0.51	−0.69	1.72	−0.57	−0.82	0.62	0.14	3.55	−0.16	1.20	−0.93
S19	0.34	−0.93	1.31	−1.39	−1.73	0.11	−0.21	2.40	−0.50	0.22	−1.78
A1	0.52	−0.81	1.93	−0.68	−0.94	0.49	0.15	4.52	−0.19	1.12	−1.59
A2	0.66	−0.66	2.00	−0.61	−0.95	0.43	0.26	4.29	−0.20	1.30	−1.01
A3	0.66	−0.52	1.67	−0.70	−1.06	0.55	0.12	2.92	−0.24	1.15	−1.19
A4	0.75	−0.57	1.85	−0.72	−0.95	0.48	0.14	3.34	−0.20	1.09	−1.09
Average	**0.65 **	**−0.78 **	**1.99 **	**−0.78 **	**−1.09 **	**0.41 **	**0.09 **	**3.60 **	**−0.21 **	**0.92 **	**−1.29 **

**Table 5:** Correlation coefficients of SMTEs in TGR sediments.

	Li	Be	B	V	Co	Ga	Sn	Sb	Tl	Bi	Fe
Li	1.000	0.278∗	0.502∗∗	0.195	0.106	0.056	0.325∗∗	−0.046	0.555∗∗	0.173	0.100
Be		1.000	0.016	0.736∗∗	0.693∗∗	0.493∗∗	0.859∗∗	−0.152	0.629∗∗	0.792∗∗	0.423∗∗
B			1.000	0.151	0.030	−0.120	0.115	−0.078	0.287∗	0.024	0.006
V				1.000	0.921∗∗	0.578∗∗	0.828∗∗	0.282∗	0.737∗∗	0.837∗∗	0.527∗∗
Co					1.000	0.537∗∗	0.806∗∗	0.291∗	0.613∗∗	0.877∗∗	0.509∗∗
Ga						1.000	0.475∗∗	0.198	0.608∗∗	0.525∗∗	0.289∗
Sn							1.000	0.071	0.689∗∗	0.865∗∗	0.444∗∗
Sb								1.000	0.168	0.151	0.133
Tl									1.000	0.575∗∗	0.292∗
Bi										1.000	0.500∗∗
Fe											1.000

*Correlation is significant at the 0.05 level (2-tailed).

∗∗Correlation is significant at the 0.01 level (2-tailed).

**Table 6:** Component pattern matrix for varimax rotated PCA analysis for SMTEs.

Elements	Component		
	PC1	PC2	PC3
Li	0.149	**0.863**	−0.075
Be	**0.876**	0.123	−0.333
B	−0.062	**0.843**	−0.004
V	**0.913**	0.171	0.203
Co	**0.909**	0.030	0.201
Ga	**0.669**	−0.050	0.222
Sn	**0.903**	0.217	−0.094
Sb	0.128	−0.037	**0.956**
Tl	**0.692**	0.533	0.147
Bi	**0.925**	0.044	0.006
Fe	**0.590**	−0.037	0.079
Explained variance in %	49.23	16.71	10.89
